# Impact of the COVID-19 pandemic on behavioral changes in healthcare workers in Italy

**DOI:** 10.3389/fpubh.2024.1335953

**Published:** 2024-02-07

**Authors:** Vincenza Sansone, Grazia Miraglia del Giudice, Giorgia Della Polla, Italo Francesco Angelillo, The Collaborative Working Group, Pasquale Di Girolamo Faraone

**Affiliations:** Department of Experimental Medicine, University of Campania “Luigi Vanvitelli”, Naples, Italy

**Keywords:** attitude, behaviors, COVID-19, face mask, hand sanitization, healthcare workers, Italy, vaccination

## Abstract

**Introduction:**

During the COVID-19 pandemic, adherence to wearing face mask and washing hands procedures and achieving high COVID-19 vaccine coverage among healthcare workers (HCWs) were essential to minimize morbidity and possible death and limit the transmission of the virus. The objectives of the cross-sectional survey were to explore the influence of COVID-19 on the use of preventive measures and vaccination willingness among HCWs in the southern part of Italy and the associated factors.

**Methods:**

The survey was carried out from 15 June 2023 to 15 July 2023 among 521 HCWs who worked in three randomly selected public hospitals. All data were collected through a self-administered questionnaire.

**Results:**

HCWs had a positive change in the use of preventive measures if they did not often/always use them before the pandemic, but they are using in the current epidemiologic context and they were willing to use in a future epidemic situation. A positive change in the adherence to face mask-wearing (24.6%) was more likely among those with at least 5 years of university degree, nurses/midwives, and who had worked in COVID-19 wards. A positive change in alcohol-based hand rubbing (3.1%) was more likely in HCWs in Emergency/Critical/Infectious Diseases wards compared with medical wards. HCWs who were more likely to believe that the COVID-19 vaccine should be mandatory for them (58.1%) had at least 5 years of university degree, in Emergency/Critical/Infectious Diseases wards compared with surgical and medical wards, had received more than three doses of this vaccine, were more concerned to get infected during their activity, and had received information from scientific journals. HCWs more willing to receive the COVID-19 vaccine every year (39.8%) were males, physicians, those in Emergency/Critical/Infectious Diseases wards compared with medical wards, who had received more than three doses of this vaccine, who believed that this vaccine should be mandatory for HCWs, and who needed additional information.

**Discussion:**

The survey showed that the COVID-19 pandemic had an impact on the use of preventive measures among HCWs, not necessarily for the improvement or increase. Educational messages on the importance of these measures regarding the promotion and recommendation of the vaccine need to be investigated and applied among HCWs in order to reduce vaccination gaps and the spread of the infection.

## 1 Introduction

It is well known that the coronavirus disease 2019 (COVID-19) pandemic has resulted in a global public health emergency, and worldwide there have been 771 million confirmed cases and 7 million deaths (1). Several statewide unprecedented containment public health measures have been implemented in response to the pandemic to reduce its impact and limit the spread of the infection. These measures included lockdowns, social distancing, mobility restrictions, temporary suspension of non-essential activities, isolation, quarantine, wearing facial masks, hand hygiene, and vaccination (2).

During the COVID-19 pandemic, healthcare workers (HCWs) have been identified as a group at an elevated risk of acquiring SARS-CoV-2 infection (3, 4) and, consequently, a high-priority group for the vaccination. There is ample evidence showing that in the hospital settings, adherence to infection prevention and control procedures, such as wearing face mask and washing hands, and achieving high COVID-19 vaccine coverage were essential to minimize morbidity and possible death and limit the transmission of the virus to other people (5, 6). However, use of face mask among HCWs during COVID-19 pandemic has been related to adverse events such as dermatitis, headache, allergy, atopy, facial itch, acne, and rash, and alcohol-based hand rub has been associated with hand eczema (7–10).

Currently, in Italy, wearing face mask in hospital is mandatory, mainly in wards with frail, older adults, or immunosuppressed patients, whereas there are no indications regarding the vaccine campaign against COVID-19 in HCWs.

While it is clear that HCWs have widely applied infection prevention and control procedures, limited information is available on the changes in wearing face mask, hand hygiene, and COVID-19 vaccination according to the pattern of the COVID-19 among the HCWs (11–13). It is imperative to fill this identified critical gap in the existing published literature. Therefore, the objectives of this current survey were to explore how the COVID-19 has influenced the use of preventive measures during the working activity and the willingness to receive COVID-19 booster vaccination among a sample of HCWs in Italy and identify the associated factors.

## 2 Materials and methods

### 2.1 Setting and survey population

The cross-sectional survey was carried out from 15 June 2023 to 15 July 2023, as part of a large research project about the preventive measures toward COVID-19. The source population included all 3,000 HCWs who worked in three randomly selected public hospitals, one teaching and two non-teaching, located in the Campania region, southern part of Italy. The sample included 480 HCWs who had been selected by a simple random sampling technique assuming that 50% of the study population would intend to wear the face mask during their activity, a 95% confidence interval, a margin of error of 5%, and a response rate of 80%.

### 2.2 Data collection

The research team asked the health director of each hospital for the permission to conduct the survey. After the approval, the team identified in each ward an HCW to distribute the questionnaire to the HCWs, who were randomly selected among those present at that moment in each ward, and collect the filled questionnaires within an envelope to maintain anonymity. The questionnaire contained a brief introduction about objectives, procedures, confidentiality, and anonymity of the survey, that the participation was voluntary, the information provided was used only for research purposes, and the participants were able to withdraw at any moment. HCWs gave their informed consent to participate by handing in the questionnaire. No incentives were given to the participants to complete the questionnaire.

### 2.3 Survey development

All data were collected through a self-administered questionnaire prepared by the research team, adapted and modified from previously published studies of the research group (14, 15). The questionnaire required 5–10 min to complete, and it aimed to collect information regarding sociodemographic, anamnestic, and professional characteristics, including gender, age, relationship status, education, professional role, duration of employment, working ward, and previous COVID-19 infection. Attitudes regarding COVID-19 have been measured with a ten-point Likert-type scale where the maximum score of “10” was assigned for the most concerned and “1” for the least concerned or with “yes” or “no” or “do not know” response options. Respondents were asked to report their behavior for the main preventive measures (wearing a face mask and alcohol-based hand rub) during their working activity before the pandemic began (March 2020), during the current epidemiologic context, and their willingness in a possible future epidemic situation. Participants were given five frequency response options from “always” to “never” for the behavior and “yes” or “no” or “do not know” for the willingness. Source(s) from which they received information related to public health measures regarding COVID-19 prevention and whether they would like to get additional information have been explored. The questionnaire was tested in a pilot survey among a group of 20 HCWs randomly selected from the source population to determine its comprehensibility, face validity, and estimated completion time, which led to refining of a few items to enhance the questionnaire. The survey responses from these first 20 respondents were not included in the overall analyses.

### 2.4 Ethics

The study protocol, the data collection instrument, and the consent form were approved by the Ethics Committee of the Teaching Hospital of the University of Campania “Luigi Vanvitelli” (code 0017091/i).

### 2.5 Statistical analysis

Descriptive statistics were used to analyze the participants' characteristics and responses to the different questions. Means with standard deviations and median were used for all continuous variables, whereas frequencies were used for the categorical variables. Then, univariate analysis was applied by using chi-square test or Student's *t*-test, respectively, to assess the association between categorical and continuous variables with the outcomes of interest. Variables that attained a *p* ≤ 0.25 in the univariate analysis were included in the respective multivariate logistic regression models, and the significant level of the *p*-value for the inclusion and elimination of the variables in the models was set at 0.2 and 0.4, respectively. Multivariate logistic regression models were constructed to examine which of the different characteristics were significantly related to these outcomes of interest: face mask-wearing during their working activity (Model 1), alcohol-based hand rub behavior during their working activity (Model 2), belief that COVID-19 vaccine should be mandatory for the HCWs (Model 3), and willingness to receive COVID-19 vaccine every year (Model 4). In Models 1 and 2, the outcome variables were coded so that HCWs who did not often/always use them before the pandemic but they are using them in the current epidemiologic context and they were willing to use in a future epidemic situation versus all other HCWs. The following independent variables of interest were tested in the univariate analysis because they were potentially related to all outcomes: gender (male = 0; female = 1), age, in years (continuous), marital status (unmarried/separated/divorced/widowed = 0; married/cohabitant = 1), having at least one chronic medical condition (no = 0; yes = 1), at least 5 years of university degree (no = 0; yes = 1), professional role (nurse/midwife = 0; physician = 1), working ward (Emergency/Critical/Infectious Diseases = 1; Surgical = 2; Medical = 3), having worked in a COVID-19 ward (no = 0; yes = 1), having been infected by SARS-CoV-2 (no = 0; yes = 1), having assisted a patient infected by SARS-CoV-2 (no = 0; yes = 1), number of COVID-19 vaccine doses received (1 = < 3; 2 = 3; 3 = > 3), concern of getting infected by SARS-CoV-2 during the working activity (continuous), scientific journals as a source of information about COVID-19 preventive measures (no = 0; yes = 1), and need for additional information about COVID-19 preventive measures (no = 0; yes = 1). In the variable working ward, the medical category includes every ward except Emergency/Critical/Infectious Diseases wards and Surgical wards. Moreover, the independent variable belief that COVID-19 vaccine should be mandatory for HCWs (no/do not know = 0; yes = 1) was included in Model 4, and the independent variable belief that wearing a face mask should be mandatory for HCWs (no/do not know = 0; yes = 1) in Models 3 and 4. To interpret the final multivariate regression models, odds ratios (OR) and their corresponding 95% confidence intervals (CI) were provided. For all analyses, *p* ≤ 0.05 were considered statistically significant. The data collected were analyzed using the STATA 18 software.

## 3 Results

Of the 586 HCWs approached, 521 consented to answer the questionnaire, giving a response rate of 88.9%. The main sociodemographic, professional, and anamnestic characteristics of the sample are presented in Table 1. The mean age was 41.7 years, most were females, 32.8% were physicians, 21.5% worked in Emergency/Critical/Infectious Diseases wards, 73.7% had been infected by SARS-CoV-2, and only 5.2% had received more than three doses of the COVID-19 vaccine.

**Table 1:** Main sociodemographic, professional, and anamnestic characteristics of the sample.

**Characteristic**	***N***	**%**
**Age, in years**	41.7 ± 12 (20–67)^*^	
**Gender**		
Female	310	61.3
Male	196	38.7
**Marital status**		
Married/cohabitant	315	62.3
Unmarried/separated/divorced/widowed	191	37.7
**Having at least one chronic medical condition**		
No	399	78.2
Yes	111	21.8
**Number of years of university degree**		
< 5	325	62.6
> 5	194	37.4
**Professional role**		
Nurse/midwife	349	67.2
Physician	170	32.8
**Length of working activity, in years**	13.4 ± 11.9 (1 month-43 years) ^*^	
**Working ward**		
Medical	312	60
Emergency/Critical/Infectious Diseases	112	21.5
Surgical	96	18.5
**Having worked in a COVID-19 ward**		
Yes	262	51.3
No	249	48.7
**Having been infected by SARS-CoV-2**		
Yes	384	73.7
No	137	26.3
**Having assisted at least a patient infected by SARS-CoV-2**		
Yes	416	80.8
No	99	19.2
**Number of COVID-19 vaccine doses received**		
< 3	59	11.5
3	429	83.3
>3	27	5.2

Number for each item may not add up to total number of study population due to missing value.

^*^Mean ± Standard deviation (range).

Respondents showed a low level of concern of getting infected by SARS-CoV-2 during their working activity, with a mean value of 4.6 measured on a 10-point Likert-type scale. Almost two-thirds (63.7%) agreed that wearing a face mask should be mandatory for HCWs, while 28.9% did not agree and 7.4% were uncertain.

Participants' behaviors and attitudes regarding the two main measures of prevention (wearing a face mask and alcohol-based hand rub) during their working activity before the COVID-19 pandemic (March 2020), during the current epidemiologic context, and their willingness in a future COVID-19 epidemic situation were explored. Less than half (42.2%) referred that they wore a face mask during their working activity before the pandemic, 76.9% currently wear it, and 64.7% were willing to wear it in a future COVID-19 epidemic. Almost one-fourth of the HCWs (24.6%) did not often/always wear a face mask during their working activity before the pandemic, are wearing it in the current epidemiologic context, and were willing to wear in a future COVID-19 epidemic situation. The results of the multivariate logistic regression analysis, examining the relationship between several variables and the two outcomes of interest, are presented in Table 2. This result was more likely to be observed among HCWs with at least 5 years of university degree, nurses/midwives, and those who had worked in COVID-19 wards (Model 1). Regarding the alcohol-based hand rub, 93.6% did it before the pandemic, 88% currently apply it, and 69.5% were willing to do it in a future COVID-19 epidemic situation. Only 3.1% of the HCWs who did not often/always apply alcohol-based hand rub in their working activity before the pandemic are doing in the current epidemiologic context and were willing to do in a future COVID-19 epidemic situation. This was more likely to be observed among HCWs working in Emergency/Critical/Infectious Diseases wards compared with those in the medical wards (Model 2 in Table 2). Moreover, only 3.1% of HCWs had a positive change for both preventive measures.

**Table 2:** Results of the multivariate logistic regression analysis showing determinants of the different outcomes of interest.

**Variable**	**OR**	**SE**	**95% CI**	***p***
**Model 1. Positive change in the adherence to face mask-wearing during their working activity**				
**Log likelihood** = −**237.03**, χ^2^ = **41.67 (7 df)**, ***p***<**0.0001**				
Having worked in a COVID-19 ward	2.57	0.66	1.55–4.27	< 0.001^*^
Nurses/midwifves	0.22	0.11	0.08–0.57	0.002^*^
Having at least 5 years of university degree	2.85	1.33	1.14–7.14	0.025^*^
Females	1.59	0.39	0.99–2.57	0.054
Working ward				
Emergency/Critical/Infectious Diseases	1°			
Surgical	0.56	0.21	0.27–1.15	0.116
Medical	0.78	0.22	0.45–1.36	0.385
Scientific journals as source of information about COVID-19 preventive measures	0.72	0.18	0.44–1.19	0.199
**Model 2. Positive change in the adherence to alcohol-based hand rub during their working activity**				
**Log likelihood** = −**57.47**, χ^2^ = **17.89 (4 df)**, ***p*** = **0.0013**				
Working ward				
Emergency/Critical/Infectious Diseases	1°			
Medical	0.15	0.09	0.05–0.51	0.002^*^
Surgical	0.13	0.13	0.16–1.01	0.051
Scientific journals as source of information about COVID-19 preventive measures	0.40	0.19	0.16–1.04	0.061
Younger	0.98	0.24	0.93–1.02	0.323
**Model 3. Belief that COVID-19 vaccine should be mandatory for the HCWs**				
**Log likelihood** = −**301.43**, χ^2^= **44.82 (8 df)**, ***p***<**0.0001**				
Having at least 5 years of university degree	2.21	0.49	1.43–3.41	< 0.001^*^
Working ward				
Emergency/Critical/Infectious Diseases	1°			
Surgical	0.47	0.15	0.25–0.86	0.015^*^
Medical	0.58	0.15	0.35–0.96	0.034^*^
Higher concern of getting infected by SARS-CoV-2 during the working activity	1.09	0.42	1.01–1.17	0.022^*^
Number of COVID-19 vaccine doses received				
>3	1°			
3	0.22	0.14	0.06–0.77	0.018^*^
< 3	0.28	0.19	0.71–1.09	0.066
Scientific journals as source of information about COVID-19 preventive measures	1.53	0.33	1.01–2.32	0.047^*^
Unmarried/separated/divorced/widowed	0.69	0.14	0.46–1.03	0.069
**Model 4. Willingness to receive COVID-19 vaccine every year**				
**Log likelihood** = −**238.15**, χ^2^= **114.91 (10 df)**, ***p***<**0.0001**				
Belief that the COVID-19 vaccine should be mandatory for HCWs	3.85	0.94	2.38–6.21	< 0.001^*^
Need for additional information about COVID-19 preventive measures	3.18	0.83	1.91–5.31	< 0.001^*^
Physicians	2.62	0.66	1.59–4.31	< 0.001^*^
Number of COVID-19 vaccine doses received				
>3	1°			
3	0.15	0.91	0.04–0.49	0.002^*^
< 3	0.16	0.11	0.04–0.62	0.008^*^
Working ward				
Emergency/Critical/Infectious Diseases	1°			
Medical	0.43	0.12	0.25–0.75	0.003^*^
Surgical	0.63	0.22	0.31–1.27	0.194
Males	0.63	0.15	0.41–0.99	0.049^*^
Unmarried/separated/divorced/widowed	0.72	0.17	0.45–1.13	0.153
Higher concern of getting infected by SARS-CoV-2 during the working activity	1.06	0.49	0.96–1.16	0.965

°Reference category.

^*^Statistically significant result.

More than half (58.1%) believed that COVID-19 vaccine should be mandatory for HCWs, whereas almost one-third (31.7%) and 10.2% did not believe that it should be mandatory and were uncertain, respectively. HCWs with at least 5 years of university degree, those working in Emergency/Critical/Infectious Diseases wards compared with HCWs in the Surgical and Medical wards, who had received more than three doses of the COVID-19 vaccine compared with those who had received only three doses, who were more concerned to get infected by SARS-CoV-2 during their activity, and who had received information from scientific journals about COVID-19 preventive measures were more likely to believe that the COVID-19 vaccine should be mandatory for HCWs (Model 3 in Table 2).

Only 39.8% of the respondents expressed the intention to accept a COVID-19 vaccine every year, 49% had no intention, and 11.2% showed uncertainty. Among those who were willing, the majority (76.8%) would receive only one dose every year, 20.3% and 2.9% would receive up to two and three doses, respectively. In the multivariate logistic regression analysis, six variables were found to be associated with HCWs' willingness to receive a COVID-19 vaccine every year. Males, physicians, those working in Emergency/Critical/Infectious Diseases wards compared with HCWs in the Medical wards, who had received more than three doses of the COVID-19 vaccine compared with those who had received three or less doses, who believed that the COVID-19 vaccine should be mandatory for HCWs, and who needed additional information about COVID-19 preventive measures were more willing to receive a vaccine against COVID-19 every year (Model 4 in Table 2).

Almost all HCWs declared to have received information about COVID-19 preventive measures (97.8%), and the most reported sources used were scientific meetings/conferences (53.3%), followed by Internet (47.5%), scientific journals (35.4%), and colleagues (31.6%). Slightly more than one-fourth (26.4%) needed to receive additional information about COVID-19 preventive measures.

## 4 Discussion

The current survey, that is part of a large research activity regarding the preventive measures toward COVID-19 including its vaccination among different groups of individuals, focuses on behavior and attitudes among Italian HCWs and provides several interesting and useful insight.

First, the prevalence of the HCWs wearing face mask was higher in the current epidemiologic context, comparing between before the pandemic and a possible future epidemic situation. Almost one-fourth of those who did not wear a face mask before COVID-19 pandemic are wearing and will wear it in a possible future epidemic situation, and the alcohol-based hand rubbing was widely used, regardless of the pandemic. However, 3.1% of HCWs who did not use alcohol-based hand rubbing before the pandemic had a positive change, doing it in the current epidemiological context and willing to do it in a possible future epidemic situation. Analyzing the behaviors for both preventive measures, only 3.1% of HCWs had a positive change. These findings underlined that the pandemic has determined an improving in the use of these preventive measures, such as reported in other studies describing an enhancing in preventive measures application during pandemic among HCWs (11, 12). This has an important impact on infection prevention in healthcare settings since wearing a face mask and alcohol-based hand rubbing are two of the most useful measures to reduce the transmission of infections among HCWs (16, 17).

Second, the willingness to accept a COVID-19 vaccine every year (39.8%) was very low. This finding is of great concern, given the well-established observation that HCWs are at higher risk of severe SARS-CoV-2 acquisition than general population and also because in Italy this vaccination is recommended and free of charge also for this group. The observed value was lower than the willingness to receive the second booster dose (52.6%) of HCWs in the same geographic area (18) and a future dose of vaccine in other countries with values of 58.2% (19) and 76.5% (20). However, more than half of the participants (58.1%) believed that COVID-19 vaccine should be mandatory for the HCWs. This data are in line with those reported in previous studies where the mandate for HCWs is largely supported (21, 22), although lower values have been observed in France (35%) (23), US (35%) (24), and UK (18%) (25). It was described that mandatory vaccination does not represent an incentive for HCWs to get vaccinated, and in some cases, it seems to be associated with a decrease in vaccine adherence (21). It is interesting to observe that those who believed that the COVID-19 vaccine should be mandatory for HCWs were more willing to receive a vaccine against COVID-19 every year. This should be considered when mandatory vaccination policies are developed to promote the uptake of COVID-19 dose. Moreover, the willingness was also more likely to be found in males, physicians, those who had received more than three doses of the COVID-19 vaccine, and those who believed that this vaccination should be mandatory for HCWs.

Third, the results of the multivariate logistic regression analysis showed that several characteristics were independently associated with the different outcomes of interest. Of the sociodemographic characteristics, only the educational level was associated with the change in behaviors and supporting COVID-19 mandatory vaccine. Indeed, the HCWs with a higher education had a positive change in wearing face-mask. This is similar to the results observed in the literature among the general population (26, 27) but not consistent with the studies on HCWs, showing that higher level was associated with worse compliance in personal protective behaviors (13, 28). Regarding the professional characteristics, the wards of activity and the role have been proven to be important factors associated with the outcomes. Indeed, in this survey, HCWs in Emergency, Critical Care, and Infectious Diseases wards showed a positive change in alcohol-based hand rubbing. This may be explained by the fact that in these settings there is a higher risk of infections, and, therefore, as described in the literature, HCWs have a stronger sense of self-protection, infection prevention, and control (28, 29). Moreover, HCWs in COVID-19 wards had also a positive change in face mask wearing. This finding has already been reported in the literature in the first pandemic phase (30, 31). Therefore, working in this area increased HCWs' awareness of the importance to protect the airways to avoid being infected and become a source of infection for patients and colleagues. HCWs in Emergency, Critical Care, or Infectious Diseases wards and those who had received more than three doses of the vaccine were more willing to accept a booster dose and more likely to support the COVID-19 mandatory vaccine. These results may be partly explained with the perceived risk of infection for themselves and their patients (25, 32). Another variable associated with the attitude toward the mandatory vaccine was the concern of getting infected by SARS-CoV-2 during their activity. Indeed, HCWs who were concerned were more likely to support the mandatory vaccine. A similar observation has been already reported in previous studies for other vaccines (30, 33–35). Nurses and midwives had a positive change in wearing face-mask than physicians, as previously reported in the literature for hand washing (36). Instead, HCWs working in Emergency/Critical/Infectious Diseases wards, physicians, and those who need more information about COVID-19 were more willing to accept a vaccine against COVID-19 every year. This confirms the results of other surveys that physicians are more vaccine-acceptant (19, 37, 38). A higher level of knowledge about vaccine effectiveness and safety than other HCWs could be a possible explanation (39).

Fourth, the sampled HCWs consulted various sources for acquiring information about COVID-19 preventive measures. However, it is necessary to underline that only approximately one-third of them had used scientific journals. This is a major concern since this source of information has a fundamental role for HCWs regarding the use of the preventive measures and on their beliefs and intention toward vaccines, on the vaccine acceptance and uptake, and on the recommendation to their unvaccinated patients. In the present study, it has been found that this source is emerged as a key positive determinant of the belief that the COVID-19 vaccine should be mandatory for the HCWs since those who used scientific journals were more likely to have this belief compared with those who relied on other sources. Moreover, many studies on HCWs conducted in different countries showed that when scientific journals were used as source of information about vaccinations, HCWs were more likely to have a higher level of knowledge, more appropriate behaviors with high uptake, and a more positive attitude toward the intentions to receive and make the recommendation to their patients (15, 18, 40–45). This association could be attributed to the fact that there is an extensive amount of scientific literature regarding the impact of COVID-19 with the related cases and deaths worldwide among HCWs, and that their vaccination was a fundamental part of the infection control strategies, providing self-protection and indirect protection to their patients with the reduction in the spread of the disease. HCWs are pivotal and it therefore becomes imperative that they have a higher access to scientific journals.

The present survey has some potential methodological limitations that should be taken into consideration when interpreting the results. First, the cross-sectional design limits the possibility to determine a cause-effect relationship of the measured associations between the explanatory variables and the outcomes of interest. Second, the recruitment of the sample was conducted in hospitals located in one region of the country; therefore, it is possible that the survey findings may not be generalized to the whole population of HCWs across Italy. Third, information was gathered through a self-administered questionnaire and could not be verified based on direct observation, and this may not provide a reliable picture of actual behavior, leading to a potential recall bias. Fourth, for the attitude regarding the COVID-19 booster vaccination, HCWs may have answered questions in a socially desirable way. However, these risks are reduced with the anonymous questionnaire, and no identifying data are collected. Fifth, the survey did not explore HCWs' perception regarding the role of preventive measures in promoting health or reducing the risk of being diagnosed by COVID-19. In addition to these described limitations, we are confident that the results give relevant and valuable information on attitudes and behavioral changes of HCWs in Italy after the COVID-19 pandemic.

In conclusion, the results of this survey showed that the COVID-19 pandemic has determined a change in the HCWs' behaviors with an increasing use of preventive measures during their activity, although the willingness to accept a COVID-19 vaccine every year was very low. Given the crucial role of HCWs in order to reduce the spread of the infection, strategic educational messages to HCWs should be implemented on the importance of the preventive measures and for the promotion and recommendation of the additional doses of COVID-19 vaccine in order to reduce vaccination gaps. Moreover, information strategies should be coupled with vaccination campaigns in the workplace, indeed getting vaccine more conveniently for HCWs determine a higher adherence. Research is needed to understand if HCWs' behaviors have changed after the pandemic, such as the use of swabs for patients' admission in hospital, isolation or quarantine in hospital in the case of fever or respiratory symptoms, and if COVID-19 has influenced HCWs' behaviors in private life.

## Data availability statement

The raw data supporting the conclusions of this article will be made available by the authors, without undue reservation.

## Ethics statement

The study protocol, the data collection instrument, and the consent form were approved by the Ethics Committee of the Teaching Hospital of the University of Campania “Luigi Vanvitelli” (code 0017091/i).

## The Collaborative Working Group

Pasquale Di Girolamo Faraone, Health Direction, Teaching Hospital of the University of Campania “Luigi Vanvitelli”, Piazza Luigi Miraglia, 80138 - Naples, Italy; Mario Massimo Mensorio, Health Direction, Sant'Anna e San Sebastiano Hospital, Via Ferdinando Palasciano, 81100 - Caserta, Italy; Mauro Muto, Health Direction, San Leonardo Hospital, Viale Europa, 80053 - Castellammare di Stabia, Italy.

## Author contributions

VS, GMdG, and GDP participated in the design of the survey, contributed to the data collection, data analysis and interpretation, and prepared the original draft. IFA the principal investigator, designed the survey, was responsible for the statistical analysis and interpretation, and wrote the article. All authors have read and agreed to the published version of the manuscript.
